# High-Sensitivity Detection of Chiro-Optical Effects in Single Nanoparticles by Four-Wave Mixing Interferometry

**DOI:** 10.1021/acsphotonics.4c01782

**Published:** 2024-12-18

**Authors:** Paola Borri, Lukas Payne, Francesco Masia, Marco Esposito, Vittorianna Tasco, Adriana Passaseo, Wolfgang Langbein

**Affiliations:** † Cardiff University School of Biosciences, Museum Avenue, Cardiff CF10 3AX, United Kingdom; ‡ CNR NANOTEC Institute of Nanotechnology, Via Monteroni, Lecce 73100, Italy; § Cardiff University School of Physics and Astronomy, The Parade, Cardiff CF24 3AA, United Kingdom

**Keywords:** chirality, nanoparticles, plasmonics, nonlinear optics, four-wave mixing

## Abstract

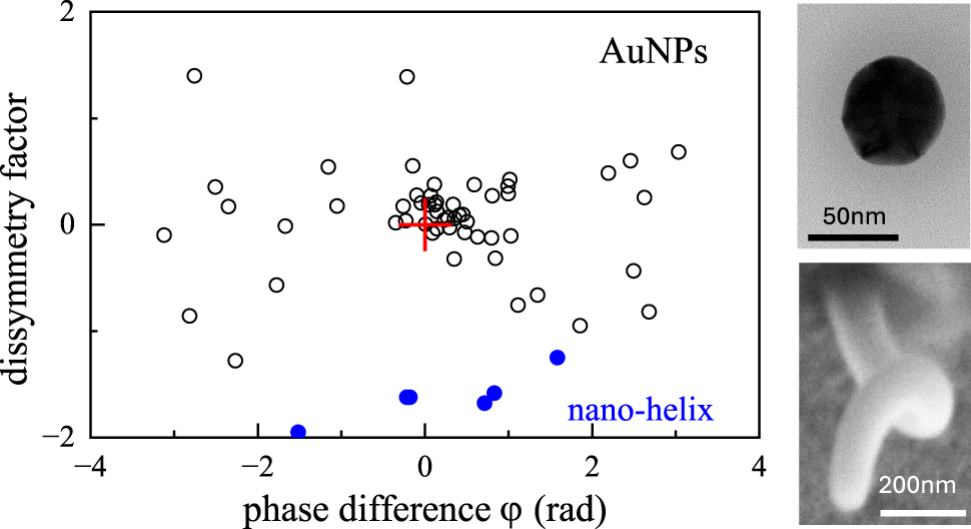

The field of chiral nanoparticles is rapidly expanding, yet measuring the chirality of single nano-objects remains a challenging endeavor. Here, we report a technique to detect chiro-optical effects in single plasmonic nanoparticles by means of phase-sensitive polarization-resolved four-wave mixing interferometric microscopy. Beyond conventional circular dichroism, the method is sensitive to the particle polarizability, in amplitude and phase. First, we demonstrate its application on single chiral nanohelices fabricated by focused ion beam induced deposition. We examined the combination of detected fields, which measures the particle polarizability, and showed that this is a sensitive reporter of chirality, providing dissymmetry factors (*g*_α_) impressively approaching unity. We then applied the method to a set of individual small gold nanoparticles near the dipole limit (60 nm nominal size), having correspondingly small chiral effects from the intrinsic lattice defects and nonperfectly spherical shape. We find that *g*_α_ is randomly distributed in the population, consistent with its nondeterministic origin, but again exhibits remarkably high values, an order of magnitude higher than those obtained using conventional light absorption. Considering the importance of chiral plasmonic nanoparticles in fields ranging from catalysis to metamaterials, this technique offers a powerful way to quantify chiro-optical effects at the single particle level with unprecedented sensitivity.

## Introduction

Measuring the chirality of single nano-objects has been attracting considerable interest in recent years. Chirality is defined as the lack of mirror symmetry (i.e., an object cannot be superposed on its mirror image by any combination of rotations and translations) and is a ubiquitous property observed in naturally occurring and artificially fabricated nanoparticles. Biomolecules such as DNA, most sugars and several proteins are chiral, and so are many pharmaceutical compounds.^1^ Notably, despite their identical chemical composition, enantiomers (the two mirror-image isomers of a chiral molecule) can have dramatically different biological functions and toxicities. Indeed, chirality is highly relevant for drug development, as single enantiomer drugs are often more efficient than their racemic mixtures. More importantly, while one chirality provides a powerful medicament, the other one may cause serious side effects.^2^

Conventional optical methods to measure molecular chirality detect the difference in extinction between left (LCP) and right circularly polarized (RCP) incident light, called circular dichroism (CD),^3^ and/or the polarization axis rotation acquired by linearly polarized incident light due to the phase shift associated with the sample’s circular birefringence (CB), called optical rotation.^4^ Since individual biomolecules produce extremely small CD and CB effects, in practice, these measurements are carried out using large numbers of molecules as ensemble averages. However, the requirement of large amounts of molecules is often a significant drawback, for example at the beginning of a drug development pipeline when large quantities of compounds are difficult to obtain due to limited production efficiency. Moreover, statistical information on variations within the sample distribution are invariably lost by ensemble averaging. In this context, there has been an increasing demand to develop novel approaches capable to detect chiro-optical effects from small numbers of biomolecules, eventually reaching the single molecule limit.

Recent works have discussed the use of plasmonic nanostructures to enhance weak CD effects for chiral molecule detection.^5,6^ Especially the use of chiral plasmonic nanostructures able to “twist” light in the near-field and in turn enhance chiral light-matter interaction phenomena at the nanoscale appears as a promising avenue.^7−9^ Notably, plasmonic nanostructures are also the building block of metamaterials, themselves featuring useful optical properties for advanced nanophotonic applications beyond sensing.^10,11^ Metamaterials with large rotatory power and circular dichroism in the visible spectral range can boost the development of integrated photonic circuits where they can operate, for example, as miniaturized polarization controllers, optical isolators, and circular polarizers.^12^

In view of the significant chiro-optical effects of individual chiral plasmonic nanoparticles compared to naturally occurring molecules, and considering their importance as building block of metamaterials, these nano-objects have been utilized for proof-of-principle demonstration of single particle chiral detection in recent years. Methods shown so far reported the differential absorption to incident circularly polarized light, detected via traditional extinction^13^ or photothermal microscopy,^14,15^ or the difference between LCP and RCP scattered light.^16−18^ Notably, none of these methods is phase sensitive, hence the manifestation of chirality in the form of optical rotation from a single nanoparticle has to date remained undetected. Moreover, all plasmonic nanoparticles investigated in these works were quite large in size (≥100 nm), well above the dipole limit. Measuring the chirality of single small plasmonic nanoparticles near the dipole limit remains challenging. Indeed, chirality is a nonlocal property,^19^ and its effect on the optical response is expected to decrease significantly with reducing nanoparticle size. Recently, the ensemble chirality of small (<100 nm) gold nanoparticles (AuNPs) was investigated using circular dichroism.^20^ It was found that single-crystal AuNPs have negligible chirality, while AuNPs with crystal lattice defects have a chirality increasing with nanoparticle size. However, the reported CD is an ensemble average of size-distributed AuNPs with right and left handed chirality, from which the single particle chirality cannot be extracted.

Here, we show a new technique to detect chiro-optical effects in single plasmonic chiral nanoparticles, using phase-sensitive polarization-resolved four-wave mixing (FWM) interferometric microscopy. First, we demonstrate the method on individual nanohelices exhibiting strong 3D chiro-optical effects. Beyond conventional circular dichroism, our method is sensitive to the particle polarizability, in amplitude and phase, which shows an exceptionally large chiro-optical response. Moreover, owing to the high spatial resolution and intrinsic sectioning capability of four-wave mixing microscopy, a full 3D spatially resolved characterization of single nanohelices is provided, going significantly beyond existing imaging modalities. The sensitivity of the method is then demonstrated for detection of chirality in nominally spherical single small AuNPs near the dipole limit (60 nm diameter), exhibiting correspondingly small chiral effects from the intrinsic atomic defects in the crystal structure.

### FWM Interferometry on Single Chiral Nanohelices

Four-wave mixing is a third-order nonlinear optical process, in which three incoming fields elicit a nonlinear response of the medium and a fourth light field is created.^21^ While the term FWM often refers to parametric processes, it also applies to processes involving light absorption, for example in resonance with excitonic transitions in semiconductor nanostructures.^22^ We are using a degenerate configuration with all incoming fields having the same wavelength. This allows us to selectively target the absorption resonance of a sample of interest and maximize its response (triply resonant FWM case).^21^ For example, by tuning the wavelength to the localized surface plasmon resonance (LSPR) of gold nanoparticles, we have shown that single small nanospheres of sizes in the dipole limit, down to only 10 nm diameter, can be detected with high sensitivity, selectivity and photostability.^23−27^ Notably, when implemented as a microscopy modality, the technique features subdiffraction limited spatial resolution in 3D,^23^ high localization precision,^25,27^ and background-free image contrast even in highly scattering and fluorescing environments.^26,28^

**Figure 1:**
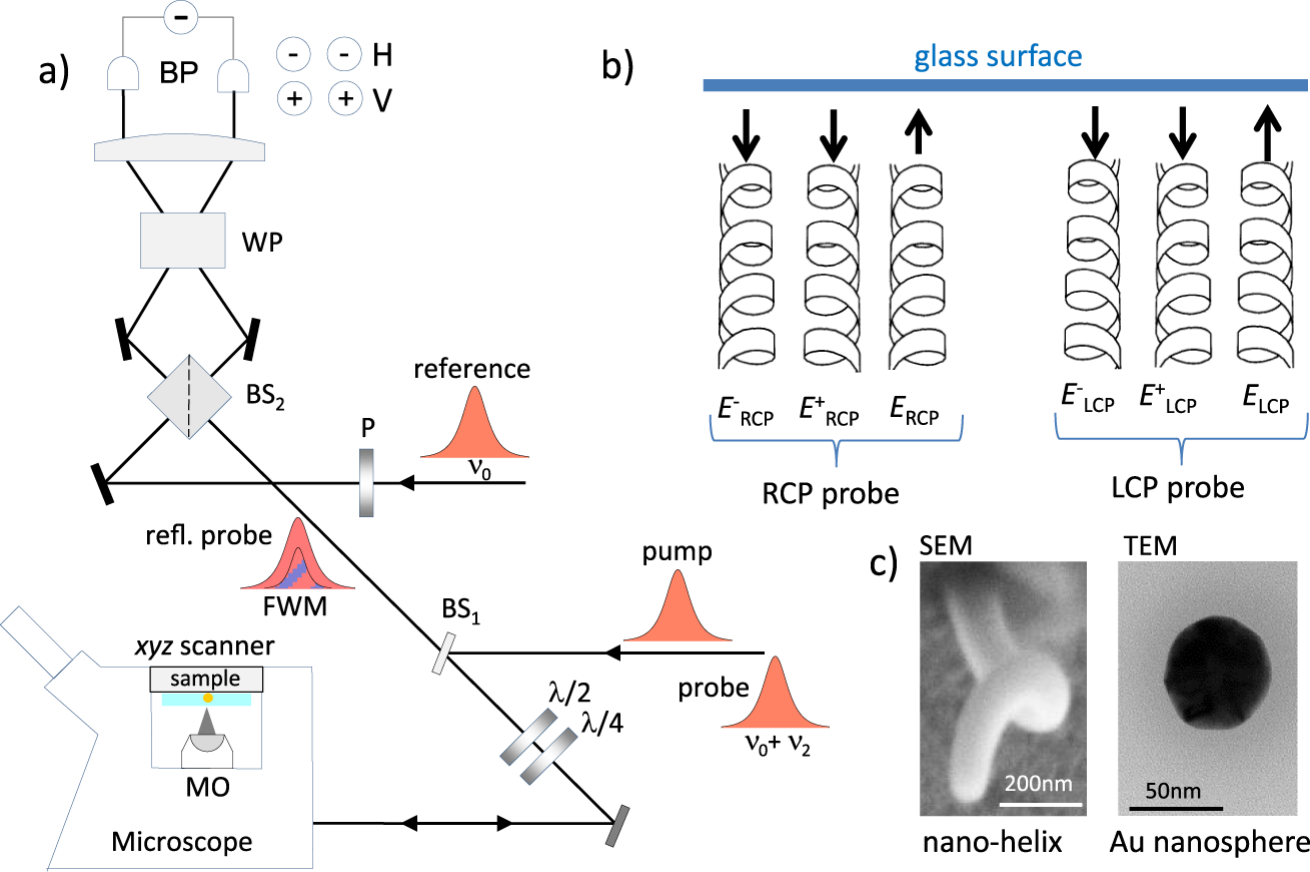
(a) Sketch of the experimental setup with interferometric detection of the FWM field and polarization-resolved configuration. Pump and probe pulses are coupled into an inverted microscope equipped with a high NA microscope objective (MO). The probe optical frequency ν_0_ is slightly upshifted by a radio frequency amount ν_2_. Incident beams are adjusted to be circularly polarized at the sample by λ/4 and λ/2 wave plates. The reflected circular polarizations are transformed into horizontal (H) and vertical (V) linear polarization by the same wave plates, and both components are simultaneously detected through their interference with a frequency unshifted reference linearly polarized at 45 deg (see text). BP: balanced photodiodes. WP: Wollaston prism. (b) The polarization of the incident probe field *E* is adjusted to be left-circular (LCP) or right-circular (RCP) at the sample. The sketch highlights that the collection is in reflection geometry, and that the light helicity is inverted upon reflection. (c) Examples of investigated nanostructures; SEM on a single nanohelix (carbon material) and TEM of a nominally spherical gold nanoparticle.

A sketch of the FWM setup is shown in Figure 1. We use a sequence of short optical pulses to increase peak field amplitudes, and thus generate large nonlinear optical effects, while maintaining low average powers at the sample. The home-built setup is similar to that used in our recent works,^25−28^ differing here only by the implementation of an image acquisition using both right and left circularly polarized incident light. Briefly, the output of an optical parametric oscillator, generating pulses of 150 fs duration centered at 550 nm wavelength with ν_L_ = 80 MHz repetition rate, is split into three beams, called pump, probe and reference. The pump excites the sample with an intensity that is on/off amplitude modulated (50% duty cycle) at ν_m_ = 0.4 MHz. The change in the sample optical properties induced by this excitation is resonantly probed by the probe pulse at an adjustable delay time after the pump pulse (all measurements in this paper are shown using 0.5 ps delay, for maximum FWM effect).^25^ Pump and probe pulses are recombined into the same spatial mode and focused onto the sample by a high numerical aperture (NA) microscope objective (MO), indexed matched to the liquid surrounding the nanoparticle investigated (see Methods for more details). The sample is positioned and moved with respect to the focal volume of the objective by scanning an *xyz* sample stage with nanometric position precision. A FWM field (proportional to the pump induced change of the reflected probe field) is collected by the same objective (epi-detection), together with the reflected probe field, transmitted by a beam splitter (BS1; 80% transmission power, 20% reflection) used to couple the incident beams into the microscope, and recombined in a nonpolarizing beam splitter (BS2; 45% transmission power, 45% reflection) with a reference pulse field of adjustable delay. The resulting interference is detected by two pairs of balanced photodiodes (BP), detecting the horizontal and vertical linear polarization separately. A heterodyne scheme discriminates the FWM field from pump and probe pulses and detects the amplitude and phase of the field. Note that heterodyne detection is particularly important in our degenerate FWM configuration with high NA objectives, since neither wavelength nor directional selection of the FWM signal is possible in this case. For heterodyne detection, the probe optical frequency is slightly upshifted by a radio frequency amount (ν_2_ = 82 MHz) and the interference of the FWM with the unshifted reference field is detected. As a result of the amplitude modulation of the pump and the radio frequency shift of the probe, this interference gives rise to a beat note with two sidebands, and replica separated by the repetition rate of the pulse train. A multichannel lock-in amplifier enables the simultaneous detection of the carrier at  and the sidebands at . Via the in-phase and in-quadrature components for each detected frequency, the amplitude (*A*) and phase (Φ) of the probe field reflected by the sample and of the epi-detected FWM field are measured. Notably, this reflection (epi) configuration offers advantages compared to a transmission geometry. First, the sample can be placed onto a user-friendly commercial inverted microscope amenable to nontransparent specimens, with wide-ranging applications. Furthermore, the reflection direction offers a “dark-field like” modality, background-free against the incident/transmitted beams, which is useful when detecting the probe beam in the experiment. Moreover, the axial position of the sample is encoded in the phase of the reflected wave, as discussed in our previous work.^25^ This axial-position sensing is not possible in transmission geometry. Such a readout can be exploited for high-precision single particle tracking, alongside chirality sensing, in future experiments.

For polarization control of the excitation and polarization-resolved detection, the following scheme was used. First, probe and pump beams, linearly polarized horizontally (H) and vertically (V), respectively, in the laboratory system, are transformed into cross-circularly polarized beams at the sample by a combination of λ/4 and λ/2 wave plates (this cross-circular pump–probe polarization configuration applies to all measurements shown in this work). The reflected probe and FWM fields collected by the same microscope objective travel backward through the same wave plates, such that the probe reflected by a planar surface returns V polarized in the laboratory system. The reference beam is linearly polarized at 45 deg (by a Glan-Taylor polarizer) prior to recombining with the epidetected signal via the nonpolarizing beam splitter BS2. A Wollaston prism vertically separates H and V polarizations for each arm of the interferometer after BS2. Two pairs of balanced photodiodes then provide polarization-resolved detection, the bottom (top) pair detecting the current difference (for common-mode noise rejection) of the V (H) polarized interferometer arms. In turn, this corresponds to detecting the co- and cross-circularly polarized components of the detected field relative to the circularly polarized probe. In the sketch in Figure 1 and in the rest of the manuscript we use the symbol + to refer to the copolarized component and – for the cross-polarized component. To generate a circularly polarized probe at the sample with either left or right helicity, we adjusted the λ/4 and λ/2 and determined the two positions that gave rise to a reflected probe field returning V polarized at the detector after reflection from a planar surface (by minimizing the H component with an intensity extinction, and hence polarization purity, better than 10^–4^, see also Section S1 for more details). For a small gold nanoparticle in the dipole limit as sample, the spatially resolved pattern of the detected field in the cross-circularly polarized component, calculated and measured in our previous work,^25^ exhibits a phase rotating as a function of the in-plane polar angle with a well-defined helicity. We have verified that the two configurations of the wave plates indeed resulted in an opposite helical rotation of this phase pattern, confirming an RCP and LCP helicity of the incident circularly polarized probe, as shown in Figure S1, and later in the paper for the measurements on small gold nanoparticles.

Figure 1c shows examples of electron microscopy images of the investigated samples. We studied single nanohelices fabricated by focused ion beam induced deposition^29^ (FIBID), nominally identical to those forming the arrays characterized in the work by Esposito et al.,^30^ exhibiting significant ensemble-averaged chiro-optical effects in the visible range. Helices have a wire diameter (WD) of 100 nm, an external diameter (ED) of 300 nm, and make a single (*N* = 1) full revolution with a vertical pitch (VP) of 500 nm, as seen in the scanning electron microscopy (SEM) image in Figure 1c. The same two material compositions reported by Esposito et al.^30^ were investigated, namely metallic platinum (Pt) based helices which exhibit a broad plasmonic resonance in the visible wavelength range, as well as dielectric carbon based (C) helices for comparison. We also investigated single gold nanoparticles of nominally spherical shape and 60 nm diameter. The transmission electron microscopy (TEM) image in Figure 1c shows that these AuNPs exhibit facets and irregularities, significantly deviating from a symmetrical spherical shape on the atomic scale.

**Figure 2:**
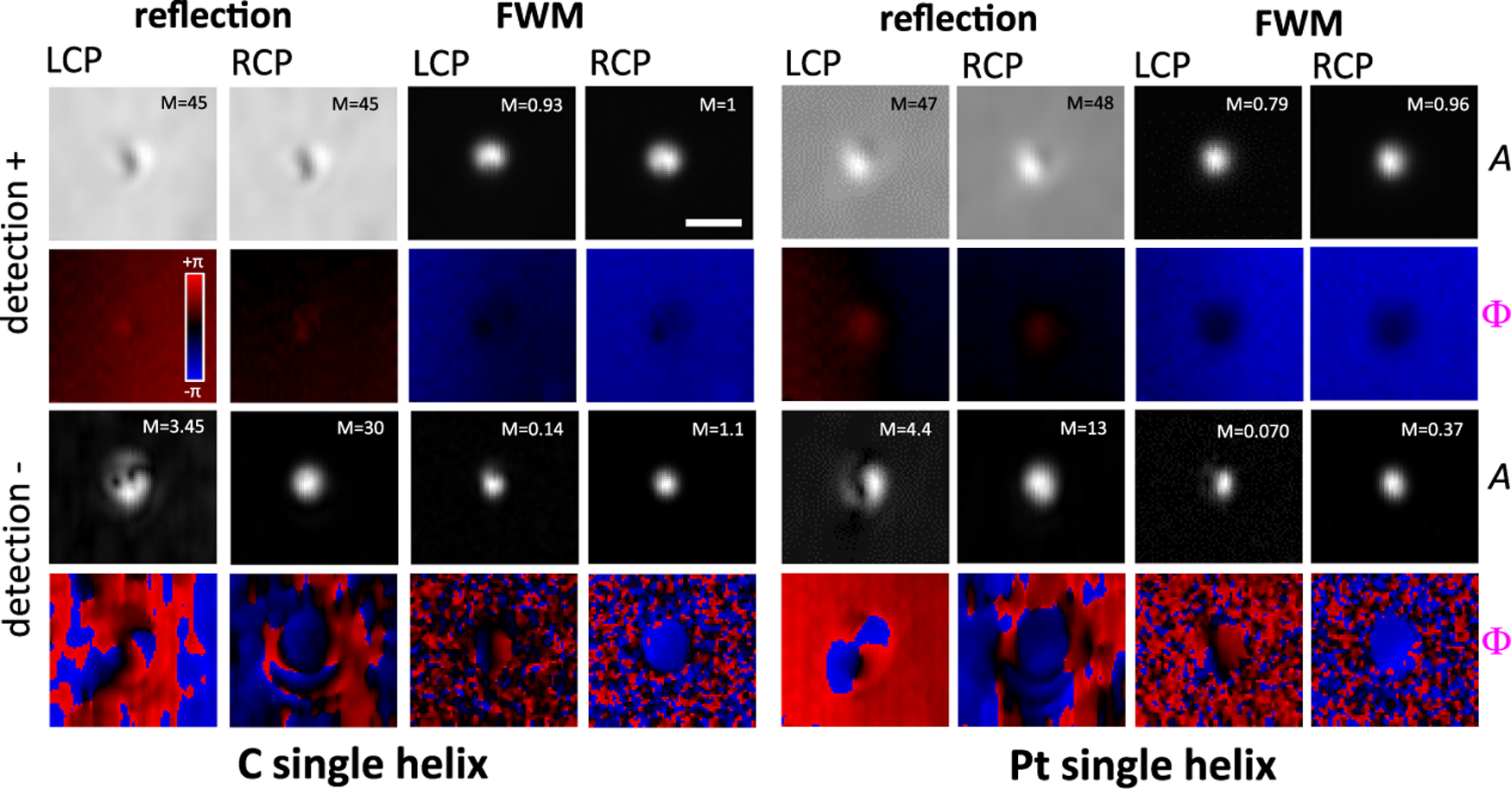
In-plane (*xy*) images of the reflected probe and FWM field patterns (amplitude *A* and phase Φ) at the optimum axial focus on a single C and Pt helix, detected as co (+) and cross-circularly polarized component (−) relative to the input circularly polarized probe, comparing the cases of an RCP and LCP helicity of the incident circularly polarized probe, as indicated. The linear gray scale is from 0 to *M* for field amplitudes (*A*), where *M* = 1 corresponds to a detected signal of 11.4 mV. Phases (Φ) are shown from −π to π in a blue-black-red scale, as indicated. Measurements were performed using a 1.27 NA water immersion objective, with a pump power at the sample of 210 μW and a probe power of 22 μW (54 μW) for the Pt (C) helix. The in-plane step size was 42 nm, and the integration time per pixel was 1 ms. Scale bar: 1 μm.

An overview of the FWM field and reflected probe field measured in amplitude and phase on a single Pt and C helix is shown in Figure 2, comparing the cases of having an RCP and LCP helicity of the incident circularly polarized probe, as indicated. Images are shown in the *xy* plane at the *z*-position of optimum focus corresponding to the maximum amplitude of the copolarized detected FWM field (). For this study, we used water as the medium surrounding the helices, and a 1.27 NA water-immersion objective for imaging (see Methods). The following main features emerge from this overview. First, the FWM detection provides a background-free contrast, as compared to the copolarized reflected probe where there is a significant background from the reflection at the interface between water and the substrate onto which the sample is fabricated (indium tin oxide—ITO coated glass). Such a clear background-free contrast is a significant advantage of FWM, enabling to distinguish single nanoparticles even when embedded within complex scattering environments.^28^ FWM also provides a smaller point-spread function (PSF), owing to the optical nonlinearity of the phenomenon.^25^ Furthermore, the presence of a strong chiro-optical effect is evident when considering the cross-polarized detected components in both reflection and FWM (see lower two rows labeled “detection −” in Figure 2). There is a significant difference between the detected amplitudes using an LCP versus RCP excitation geometry, with the LCP case always resulting in the lowest amplitudes (by nearly 1 order of magnitude in the FWM component). Notably, the LCP helicity is the one opposite to the helix geometry (see Figure 1). It is also important to point out that our setup operates in reflection. Since the helicity of an incident circularly polarized field is flipped upon reflection from a planar surface, the “detection +” field component is actually polarized with the opposite helicity relative to the incident probe. In other words “detection +” refers to light cocircularly polarized with the *reflected* probe field, and this has opposite helicity compared to the *incident* field. Conversely, the field detected cross-circularly polarized has the same helicity as the incident circularly polarized probe field (see also in sketch in Figure 1). Therefore, when detecting the cross-polarized component (indicated as *E*^–^ in Figure 1) we are sensitive to a field that either has the same helicity as the nanohelix geometry in both incident *and* reflected wave (RCP case), or is opposite to it (LCP case).

It is also interesting to note that the observed chiro-optical effects are similar between the metallic Pt helix and the dielectric C helix, suggesting a behavior dominated by the geometrical helicity. This is consistent with measurements of the time-resolved FWM dynamics versus pump–probe delay time on these helices (see Figure S2) showing a long-lived response dominated by photothermal effects in both samples, namely a heating of the surrounding water medium and consequent change of its refractive index which modulates the reflected probe. As shown by Esposito et al.,^30^ in the metallic Pt helix, the nanowire consists of Pt nanograins embedded into an amorphous carbon matrix, with a broad absorption resonance. This broad spectrum can explain the lack of a pronounced plasmonic response in the measured FWM dynamics (within the noise in the data) for the Pt helix, despite its metallic nature. This is because the pump-induced ultrafast excitation of a hot electron gas in the metal broadens and shifts the plasmonic resonance in the particle polarizability.^24^ In turn, this cannot lead to a pronounced change experienced by the probe (hence FWM), if the plasmonic resonance is already very broad in the absence of the pump. Instead, the absorption from the carbon matrix, occurring in both the C and Pt helices, gives rise to photothermal effects. The dependence of the measured FWM field amplitude on the power of the pump–probe beams is shown in Figure S3, and follows the expected third order nonlinearity.

Let us now introduce the following conceptual framework, associating the measured FWM and reflected probe fields with the particle absorption cross-section and polarizability. As demonstrated both experimentally and by comparison with simulations in our previous works,^24,25,31^ the FWM field is generated via a pump–probe mechanism, i.e., it is proportional to the pump-induced change of the reflected probe field due to the absorption of the pump field from the sample. Hence, it can be expressed as  where *F* is the FWM field, *R* is the probe reflected field, σ is the sample absorption cross-section, and η is an efficiency coefficient linking the FWM field to the sample absorption. The index *i* represents the incident probe polarization, namely *i* = L (for LCP), R (for RCP), while  = R, L denotes the pump incident polarization (which is opposite to the probe), and *j* labels the detection polarization, i.e., . For *j* = +, the reflected probe field  has the opposite helicity than the incoming probe field, hence it has interacted with both helicities in the structure. Therefore, it is reasonable to assume that the efficiency , since both helicities are probed, independent of *i*. Hence, we can extract the chiro-optical quantity  through a combined ratio of FWM and reflected probe fields, namely . Notably, since  under the same probe power conditions (see also Figure 2), the formula further simplifies into .

The other physical quantity that is measured in our experiment is the particle polarizability α (proportionally factor linking the incident light field with the particle induced dipole moment,^24,25,32^ and hence the detected field). In particular, since the reflected probe field detected cross-circularly polarized is probing the same helicity in the incident and reflected direction as discussed above, we can link the polarizability to this detected field as , assuming equal incident probe intensities in the LCP and RCP configurations. Alternatively, we can use the equality  where we have compensated the pump absorption contribution σ by taking the FWM ratio, to obtain only the probe reflection effect, and the right-hand side is intrinsically ratiometric i.e., independent of the incident intensity. Here, we also assumed that  (albeit η^–^ is related to probing one helicity only and thus could depend on helicity). However, this assumption is justified because the FWM is created by a modulation of the scalar permittivity of the material used,^24,31^ hence the resulting efficiency should not depend on the helicity.

We highlight that the absorption cross-section is proportional to the imaginary part of the dipole polarizability, hence, albeit linked, the physical quantities  and  are also different. Having access to both quantities is a strength of our FWM setup.

**Figure 3:**
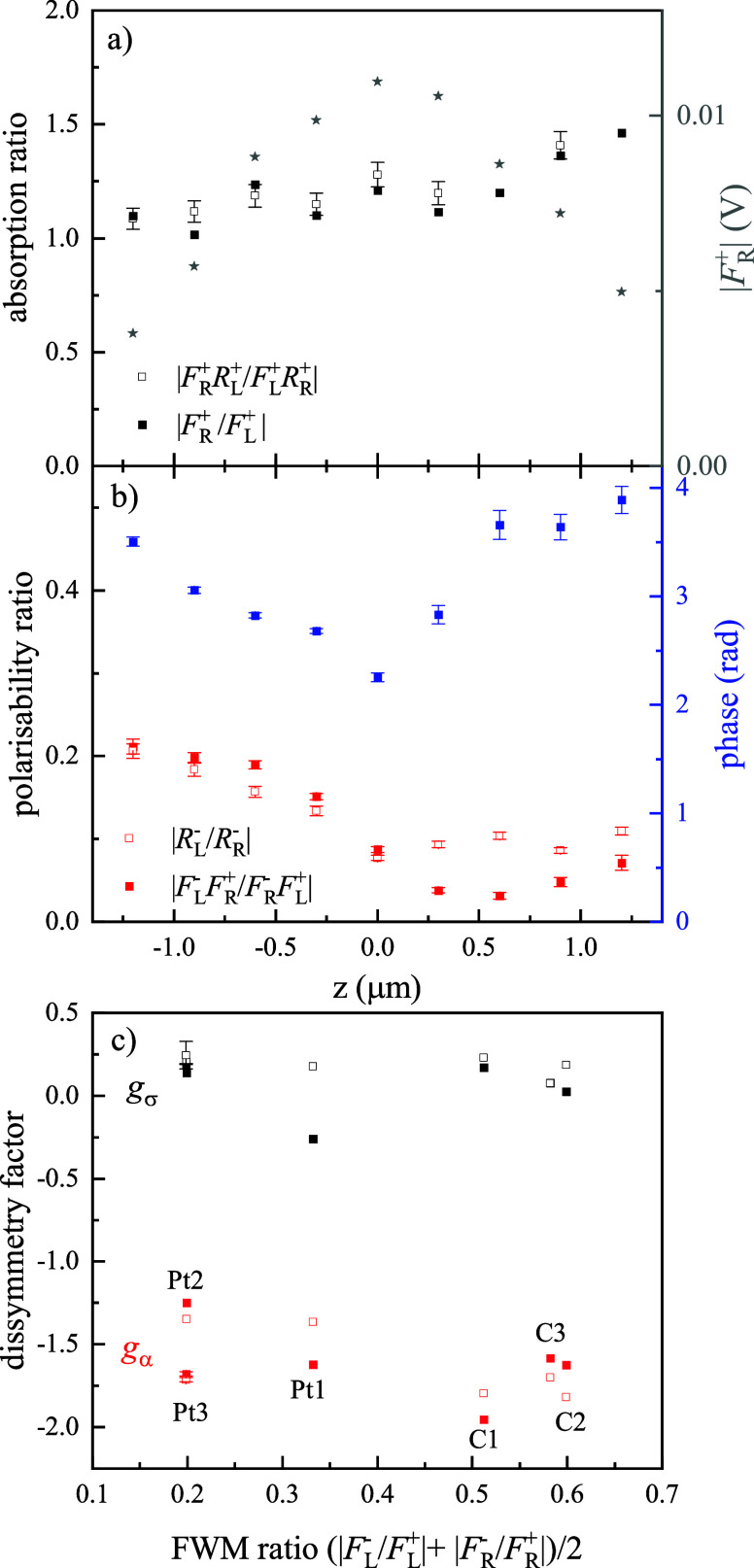
Overview of chiral quantities in single helices. (a,b) Dependence on the axial focus position *z* for the same Pt helix as in Figure 2. The star symbols with the right axis in a) show the *z*-dependence of the copolarized detected FWM field amplitude in the RCP incident probe configuration. The phase in b) refers to the polarizability ratio calculated using ; absolute phase drifts between the measurements are compensated in the ratio. Error bars are calculated as one standard deviation, from the photon shot-noise and the laser fluctuations in the experiment (see text). When not shown, errors are smaller than the size of the symbols. (c) Dissymmetry factors *g*_σ_ and *g*_α_ for all available helices (3 Pt and 3 C) measured at optimum focus versus cross to copolarized FWM amplitude ratio (average of L and R). Symbols relate the corresponding quantities used to calculate the dissymmetry factor, as per legend in (a) and (b). The C and Pt helices shown in Figure 2 correspond to C2 and Pt3. Representative error bars are shown for Pt3.

Figure 3 shows an overview of the quantities defined above, measured on single Pt and C helices. Error bars are shown, calculated by propagating the errors in each measured quantity, taking into account the photon shot-noise as well as laser fluctuations, considering that the measurements with LCP and RCP input beam helicities are carried out sequentially (see also Section S2 for details on the error propagation calculation). The top two panels display the absorption ratio  and the polarizability ratio  in amplitude and phase, for the Pt helix shown in Figure 2 measured at the *xy* location corresponding to the center of the objective point-spread function, identified as the position of maximum amplitude of the *F*^+^ field. The quantities are shown using the definitions previously introduced, either including the reflected fields or using solely the FWM fields, which give similar results and support the assumption that the efficiency coefficient η does not depend on the helicity. The dependence on the axial coordinate *z* is shown, where *z* = 0 is the plane of optimum focus (see star symbols and right axis in Figure 3a), and increasing *z* corresponds to moving the focus from the glass substrate into the helix. We notice a dependence consistent with the significant extension of the nanohelix (500 nm vertical pitch), comparable to the light wavelength, and taking into account the high axial resolution of the FWM microscope when using a high NA objective.^23^ We also observe that, while the absorption ratio is close to 1 in the focal plane, the polarizability ratio is below 0.1, with a minimum behavior near the optimum focus. In other words,  exhibits the most significant chiro-optical response, with more than an order of magnitude difference between the LCP and RCP case. The bottom panel of Figure 3 shows the dissymmetry factor, defined as  for the absorption cross-section^14^ and  for the polarizability, on nominally identical Pt and C helices at the optimum focal plane. While *g*_σ_ is around 0.2, considered a large value for a single chiral nanoparticle,^14^*g*_α_ is larger than 1 (in absolute value), which is an extraordinary chiro-optical response. Moreover, Pt and C helices exhibit similar values of dissymmetry factors, again indicating that the observed chiro-optical effect is dominated by the geometrical helicity (i.e., is not mediated by a plasmonic resonance).

The dissymmetry factor *g*_α_ introduced here should inspire the development of theoretical frameworks linking geometrical chiral particle parameters to the particle polarizability. To that end, a recent theoretical paper treated the chiro-optical response in the form of circular dichroism for a helix geometry.^33^ While this theoretical work does not explicitly introduce the particle polarizability, it derives an expression for the circular dichroism extinction coefficient ϵ_CD_ (the difference between the LCP and RCP extinction coefficients) which can be used to infer an effective chiral polarizability difference using the relationship  known from light-matter interaction principles. The theoretical work by Andrews and Tretton^33^ shows a dependence of ϵ_CD_ linearly increasing with the radius of the helix and with the helix pitch angle (in the small angle limit), providing a tool for the predictive design of nanohelices with controlled chiro-optical properties.

### FWM Interferometry on Individual Gold Nanoparticles

**Figure 4:**
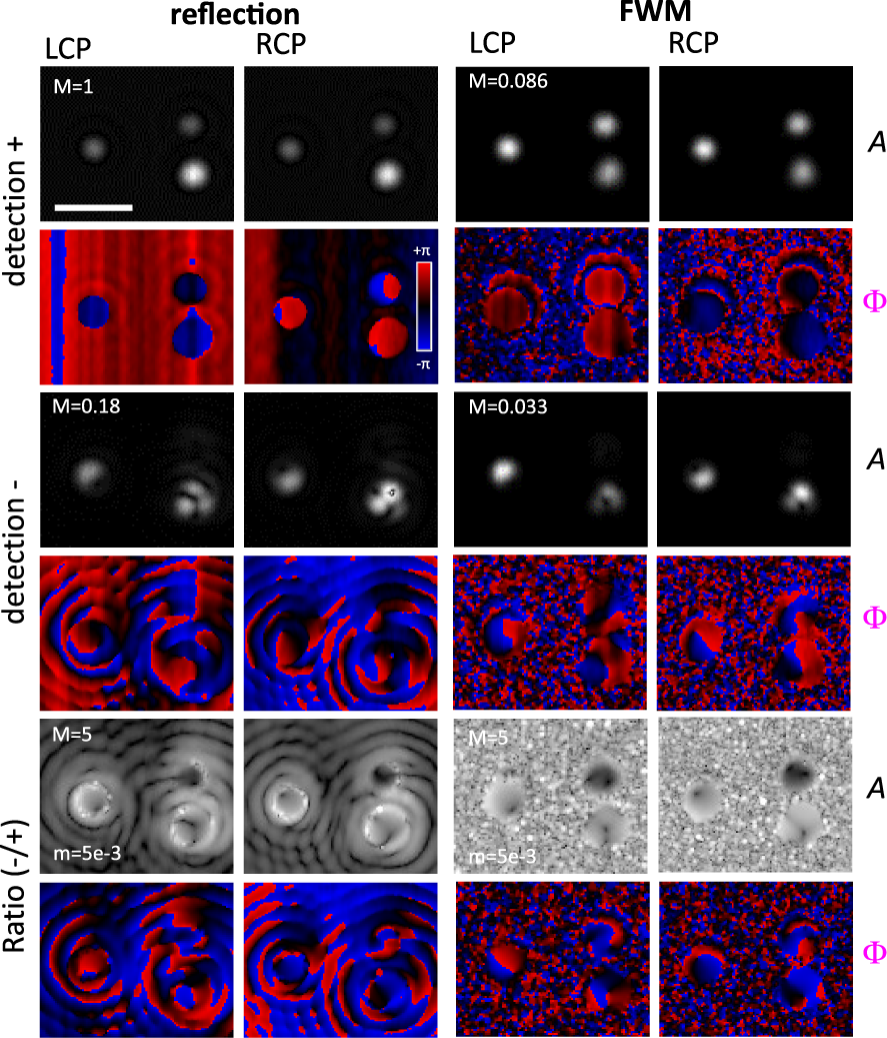
In-plane (*xy*) images of the reflected probe and FWM field patterns (amplitude *A* and phase Φ) on individual AuNPs nominally spherical with 60 nm diameter, detected as co (+) and cross-circularly polarized component (−) relative to the input circularly polarized probe, comparing the cases of an RCP and LCP helicity of the incident circularly polarized probe, as indicated. The linear gray scale is from 0 to *M* for field amplitudes (*A*). Here, *M* = 1 corresponds to a detected signal of 45 mV. Phases (Φ) are shown from −π to π in a blue–black–red scale, as indicated. The bottom row shows the ratio of the cross- relative to the copolarized detected components, on a logarithmic gray scale from *m* to *M* as indicated. Measurements were performed using a 1.45 NA oil immersion objective, with a pump (probe) power at the sample of 40 μW (20 μW). The in-plane step size was 20 nm, and the integration time per pixel was 0.5 ms. Scale bar: 1 μm.

Following from the characterization of the nanohelices, fabricated to be strongly chiral nanostructures with a well-defined geometry, we examined individual AuNPs of nominally spherical shape and 60 nm diameter, for which we expect much smaller (and randomly distributed) chiro-optical effects, due to crystal defects and shape irregularities on the atomic scale. By comparison with the nanohelices, the FWM nonlinearity measured in these AuNPs is dominated by the ultrafast heating of the hot electron gas in the metal, reaching its maximum at 0.5 ps pump–probe delay (see Figure S2), and not by photothermal effects. An example of the FWM and reflected probe field measured in amplitude and phase on selected individual AuNPs is shown in Figure 4, comparing RCP and LCP helicity of the incident circularly polarized probe, as indicated. Images are shown in the *xy* plane, and were acquired using oil index-matched to glass as the medium surrounding the AuNPs and a 1.45 NA oil-immersion objective (see Methods). Small chiro-optical effects are clearly visible as different amplitudes of the cross-polarized detected reflection and FWM fields, between the LCP and RCP case. Notably, by surrounding the AuNPs with oil index-matched to glass, we are minimizing artifacts arising from symmetry-breaking reflection at the substrate. In other words, we expect the observed chiro-optical effects to be dominated by the intrinsic AuNP geometry.

**Figure 5:**
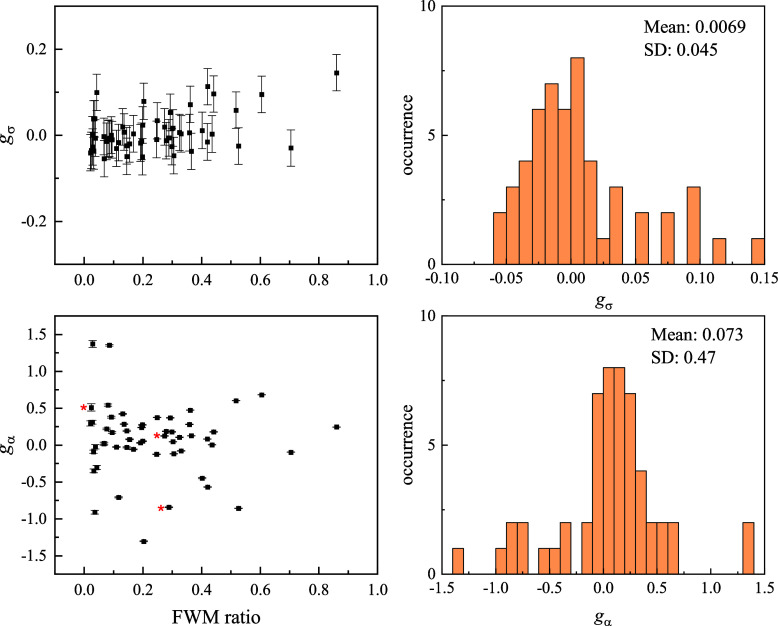
Overview of chiral quantities in single nominally spherical AuNPs of 60 nm diameter. Left: dissymmetry factors *g*_σ_ and *g*_α_ for all available AuNPs, measured at the spatial location corresponding to the center of the objective point-spread function for copolarized FWM, versus cross to copolarized FWM amplitude ratio (average of L and R). Error bars are calculated as one standard deviation, from the photon shot-noise and the relative laser fluctuations in the experiment (see text). The red stars indicate the 3 AuNPs imaged in Figure 4. Right: corresponding histograms of *g*_σ_ and *g*_α_, with indicated mean and standard deviation (SD).

As for the nanohelices, we evaluate the quantities  and  with the corresponding dissymmetry factors *g*_σ_ and *g*_α_, respectively. In total, 56 AuNPs were measured under the same excitation and detection conditions as for the example in Figure 4, and the corresponding statistical distribution of the dissymmetry factor is shown in Figure 5. For this statistical analysis, measurements were carried out by acquiring *xy* images over a large area, for which we verified that no significant focus drift had occurred during the scan (see Figure S4). Note also that for these AuNPs, the FWM ratio was found to be constant as a function of the axial position over a ∼400 nm range around the optimum focus (see Figure S5), consistent with the small size of these particles, close to the dipole limit. We measure *g*_σ_ values distributed around 0, consistent with the expectation that these nanoparticles have a much smaller chiro-optical response compared to nanohelices, and the sign of the dissymmetry is randomly distributed. Remarkably, *g*_α_ exhibits much larger values, with a mean and a standard deviation that are an order of magnitude higher than those of *g*_σ_. In other words, as observed for the nanohelices, we find that the particle polarizability ratio and its corresponding dissymmetry factor *g*_α_ is a sensitive reporter of optical chirality. As discussed previously, error bars are calculated by propagating the errors in each measured quantity, taking into account the photon shot-noise as well as laser fluctuations (see also Section S2), and are shown in Figure 5. Since *g*_σ_ is calculated using , it is significantly affected by laser fluctuations (found to be about 3%). This is because the ratios  and  only partially compensate the dependence on the incident power (the FWM field is proportional to the probe field and the pump-induced change in the particle extinction.^24^ Conversely, *g*_α_ depends solely on the FWM ratios  and  compensating laser fluctuations (for each input helicity, co- and cross-polarized FWM field are acquired simultaneously) and is affected only by the shot-noise in the experiments, which results in much smaller error bars as seen in Figure 5. Therefore, not only is *g*_α_ much larger than *g*_σ_ but is also significantly less affected by fluctuations and drifts in the measurements.

**Figure 6:**
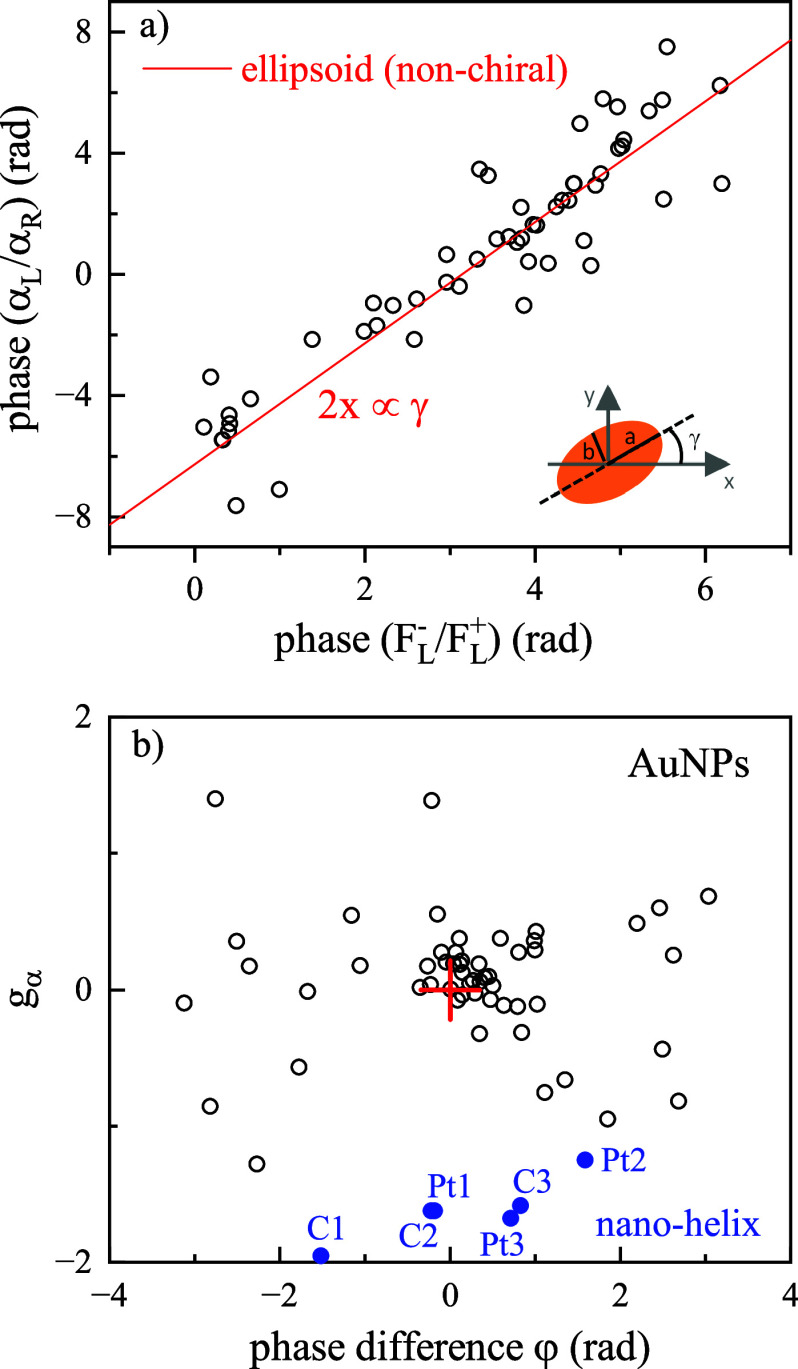
(a) Phase of the polarizability ratio  versus phase of the cross to copolarized FWM field ratio , in single nominally spherical AuNPs of 60 nm diameter (same as in Figure 5). The red line shows the expected dependence assuming a nonchiral quasi-spherical particle of ellipsoidal shape, with in-plane orientation γ as sketched in the inset. (b) Dissymmetry factor *g*_α_ versus the difference between the experimental phase of  and the phase expected from the ellipsoid model. Blue symbols are the results for the nanohelices (using the phase measured at the optimum focus *z* = 0, see Figure 3). The center cross represents the ellipsoid model case.

Furthermore, since the FWM technique is phase sensitive, we examined the phase of the polarizability ratio . This is shown in Figure 6. Note that, although our interferometer setup is not actively phase-stabilized and thus absolute phases are drifting, by examining ratiometric field quantities, hence phase differences of fields measured at the same time, we compensate for phase drifts which are present in both components. As shown in Figure 6a, some nanoparticles exhibit a very good correlation between the phase of  and the phase of the cross to copolarized FWM ratio . Using an ellipsoid model to describe quasi-spherical nonchiral AuNPs (see also discussion in Section S1), we have shown in previous works^25,27^ that the phase of  is directly related to the AuNP in-plane orientation, as sketched in the inset of Figure 6a. Note that nominally spherical AuNPs have imperfections at the nanoscale which result in nonsphericities, as evident in TEM (see Figure 1c), whereby an ellipsoid model is a reasonable first approximation to describe their shape. In this model, changing the polarization from LCP to RCP in the FWM experiment changes the sign of the FWM ratio phase. In other words, , where Φ denotes the phase. In this case, the phase of the polarizability ratio  is equal to 2. The red line in Figure 6a shows this relation. To highlight the response from chiral AuNPs deviating from this nonchiral shape-ellipsoid model, we considered the difference (φ) between the experimentally measured phase of  and the expectation from the ellipsoid model. Figure 6b shows the dissymmetry factor *g*_α_ plotted versus this phase difference φ. The cross in the center highlights the case of nonchiral particles from the ellipsoid model. In this representation, we see a group of AuNPs clustered around the cross, while other particles are distributed significantly away from it, suggesting that these particles are chiral. For reference, the values for the Pt and C nanohelices are also plotted (blue symbols), representing a strongly chiral system, well separated from the center cross. It is interesting to see, in this representation, cases where  yet the φ ≠ 0, and, vice versa, cases where *g*_α_ ≠ 0, yet , for example the nanohelices appear to group in this latter combination. We hypothesize that φ encodes an information linked to the in-plane orientation of the chirality. Nanohelices are fabricated to spiral along the *z*-axis, hence do not have an in-plane orientation component (unless fabrication imperfections occur). Conversely, AuNPs are randomly oriented, and some might exhibit a chirality direction aligned in-plane. In turn, this orientation might correspond to a small *g*_α_, if the particle shape appears achiral in its in-plane projection. A verification of this hypothesis requires a theoretical model of the light-matter interaction in a chiral particle in the nonlinear optics regime (specifically FWM), which is non trivial and beyond the scope of this work. Yet, these results show that phase-sensitive FWM interferometry provides important new metrics to separate chiral from non chiral nanoparticles, with unprecedented sensitivity.

Finally, on the same set of AuNPs, we carried out polarization-resolved optical extinction microscopy, developed by us to characterize the size and shape (specifically the in-plane asymmetry) of AuNPs described as quasi-spherical ellipsoids.^32,34,35^ This analysis was undertaken in order to determine whether there is a correlation between the measured chirality and the AuNP in-plane asymmetry. In polarization-resolved optical extinction microscopy, we use wide-field illumination and image the field of view containing all the AuNPs under investigation onto a fast sCMOS camera. By referencing against an image acquired upon laterally shifting the sample, the differential transmission is determined, from which the optical extinction cross-section of each individual AuNP in the image is quantified.^32^ To determine the nanoparticle in-plane asymmetry, measurements are carried out using a rotatable linear polarizer in the back focal plane of the condenser lens in the illumination beam path. If a AuNP is elongated in-plane, the extinction cross-section as a function of the polarizer rotation angle shows a sinusoidal dependence, the amplitude of which can be used as a reporter of the particle asymmetry (see also Methods). Results of this analysis are shown in Figure S6 and indicate no obvious correlation between *g*_α_ and the particle elongation, suggesting that the measured chirality is not linked to the particle in-plane ellipticity.

## Conclusion

In summary, we have shown that FWM interferometric microscopy offers a powerful method to detect chiro-optical effects in single plasmonic nanoparticles. Single chiral nanohelices on a planar substrate were investigated, fabricated by FIBID with metallic (using Pt) or dielectric (using C) material composition. Spatially resolved FWM and reflected probe fields were detected, polarization-resolved, in amplitude and phase, and the results were compared using left and right circularly polarized incident light. Beyond traditional circular dichroism, the technique measures the complex particle polarizability. Via appropriate combinations of the detected field components, we identified the ratiometric comparison between the co- to cross-circularly polarized FWM fields relative to the probe field, namely , to be a highly sensitive reporter of chirality, with an associated dissymmetry factor *g*_α_ approaching the maximum possible value of 2 (in absolute value). Pt and C helices exhibit similar values of dissymmetry factors, suggesting that the observed chiro-optical effect in this case is not enhanced by a plasmonic resonance, but rather determined by the geometrical helicity (notably the helices were fabricated with a vertical pitch close to the wavelength used in the experiment).

When applying the method to a set of 56 individual gold nanoparticles with 60 nm nominal diameter near the dipole limit, having faceting and crystal defects on the atomic scale, and accordingly small nondeterministic chiralities, we found that *g*_α_ was again showing a distribution of values much higher than ever reported on this type of small nanoparticles, highlighting its extraordinary sensitivity to chirality. Notably, the experimental error in *g*_α_ is affected only by shot-noise in the measurements, while laser fluctuations are compensated through FWM field ratios acquired simultaneously. Furthermore, when considering the phases of the FWM fields, we identified a phase-related component φ, allowing us to further separate chiral versus non chiral particles, in a two-dimensional (*g*_α_, φ) space. We point out that the measured FWM nonlinearity is not of photothermal nature *per se*, as shown for the gold nanoparticles. For chiral plasmonic nanoparticles exhibiting a sharp absorption spectrum such as for example gold helicoids,^36^ we expect the FWM nonlinearity to be dominated by the ultrafast heating of the hot electron gas, as opposed to the long-lived photothermal effects observed here for the nanohelices. To that end, our method does not require to embed particles in specific surrounding media.

Overall, our findings highlight the powerful capabilities of FWM interferometric microscopy as a sensitive tool to characterize the chiro-optical response of single small plasmonic nanoparticles. Notably, artificially fabricated chiral NPs hold great promise as next generation advanced functional materials for applications ranging from chiral catalysis and sensing to metamaterials and integrated nanophotonics. The methodology shown in this work could prove instrumental toward achieving a better understanding and control of the chiro-optical properties of single particles for the development of these novel materials.

## Methods

### FWM Experiments

Four-wave-mixing and reflection microscopy were performed using the setup as described by Zoriniants et al.^25^ Single nanohelices fabricated onto ITO-coated glass were immersed in deionized water, covered by a No. 1.5 coverslip and sealed with colorless nail varnish. The objective lens was 60× 1.27 NA water immersion with adjustment collar set to match the coverslip thickness. The sample is mounted with the coverslip in contact with the water immersion objective, hence the helices, attached onto the ITO-coated substrate, are facing down (see Figure 1c). Nominally spherical AuNPs of 60 nm diameter were drop cast onto a glass coverslip, immersed in silicon oil and covered by a glass slide. The objective lens was 100× 1.45 NA oil immersion. A 1.5× tube multiplier was used with both objectives, to ensure full illumination coverage of the back focal plane and thus maximum use of the objective NA.

### Wide-Field Extinction Microscopy

Wide-field extinction microscopy was performed using the setup described by Payne et al.^32^ Specifically, for the results shown in Figure S6, the illumination was provided by a 100 W halogen lamp with a color bandpass filter (Semrock) centered at 530 nm of 43 nm width. The linear polarizer in the illumination was rotated over 150 degrees in steps of 15 degrees. For referencing, the sample was laterally shifted by 2 μm. A 100× 1.45 NA oil immersion objective lens with a 1.5× tube multiplier was used. The analysis was performed as described Payne et al.,^32^ namely an extinction cross-section for the green illumination color σ_G_ was extracted and its dependence on the rotation of the polarizer angle γ_P_ was fitted using . The parameter α_G_ is a measure of the particle in-plane ellipticity. In Figure S6, the uncertainty in α_G_ is shown as error bar, obtained from repeating the fit 100 times by adding the known noise of the data.^34^

## Data Availability

Information about the data created during this research, including how to access it, is available in the Cardiff University data archive at 10.17035/d.2024.0325380380.

## Supporting Information Available

Additional experimental details and methods, including additional four-wave mixing data sets (PDF)
