# Supplementary material for: High-Sensitivity Detection of Chiro-Optical Effects in Single Nanoparticles by Four-Wave Mixing Interferometry
Source: ACS Photonics. 2024 Dec 18;12(1):392–401. doi: 10.1021/acsphotonics.4c01782 (PMC11741159; doi:10.1021/acsphotonics.4c01782)
Supplement: Supplementary file 1 — ph4c01782_si_001.pdf [file ph4c01782_si_001.pdf]

High-sensitivity detection of chiro-optical effects in single nanoparticles by four-wave mixing interferometry - Supplementary Information

Paola Borri,[1,*] Lukas Payne,[1] Francesco Masia,[1] Marco Esposito,[2] Vittorianna Tasco,[2] Adriana Passaseo,[2] and Wolfgang Langbein[3,†]

[1] *Cardiff University School of Biosciences, Museum Avenue, Cardiff CF10 3AX, United Kingdom*

[2] *CNR NANOTEC Institute of Nanotechnology, Via Monteroni, Lecce 73100, Italy*

[3] *Cardiff University School of Physics and Astronomy, The Parade, Cardiff CF24 3AA, United Kingdom*

* borrip@cardiff.ac.uk

† langbeinww@cardiff.ac.uk

S1. POLARISATION CONTROL

As stated in the paper, to generate a circularly polarised probe at the sample with either left or right helicity, we rotated the $\lambda/4$ and $\lambda/2$ waveplates and determined the two angular settings of the pair that gave rise to a reflected probe field returning V polarised and minimising the H polarised field component at the detector, after reflection from a planar sample surface. These two settings result in the two helicities of circular polarisation at the sample. More specifically, we detected the reflected field amplitude from a planar gold film coated on a glass coverslip and adjusted the $\lambda/4$ and $\lambda/2$ waveplate rotation angles to minimize its interference with the corresponding cross-polarized (H) reference field. This can be done to a precision better than 0.1% relative amplitude, but we find that the alignment drifts over the timescale of minutes to hours (likely due to beam pointing drifts), and 1% is determined as upper limit over the measurement time after adjustment. Therefore, the circular polarisation control at the sample is better than 1% as amplitude ratio or 10^{-4} as intensity extinction.

Fig. S1 shows the probe reflected field and the FWM field, as amplitude and phase patterns, on a single gold nanoparticle of nominally 60 nm diameter, for the two configurations of the waveplates giving rise to opposite circularly polarised light at the sample, corresponding to an RCP and LCP helicity of the circularly polarised probe, as indicated.

In our previous work [1] we discussed the origin of the observed amplitude and phase patterns. Assuming a perfectly spherical nanoparticle in the dipole limit, the pattern of an optical vortex of topological charge $l = 2$ is expected in the cross-polarised field components. This is due to the high NA of the objective and the vectorial nature of the field, which results in a significant cross-circularly polarized component at the focal plane forming this optical vortex, namely having a "doughnut" amplitude pattern (zero in the focus center and radially increasing away from the center), and a phase changing with twice the in-plane polar angle. Conversely, using an ellipsoidal nanoparticle model, the occurrence of a homogeneous signal region at the focus centre with two surrounding minima in the amplitude of the cross-polarised reflection and FWM field components is explained by the particle asphericity (see Fig.4 in Zoriniants *et al.* [1]). Notably, the ratio between the cross and co-polarised FWM fields at the center was shown to scale linearly with the nanoparticle ellipticity (see Fig.S5 in Zoriniants *et al.* [1]). Furthermore, the phase of the ratio was shown to be proportional

to twice the in-plane orientation angle of the ellipse. Such amplitude and phase patterns are observed in Fig. S1 for both the LCP and RCP circular polarisation configuration in the experiment, but they are not identical, hinting at chirality, as discussed in the main paper (see Fig.6a).

S2. ERROR PROPAGATION

The individual errors found in the experiments are the photon shot-noise limited errors in the FWM amplitudes $\Delta|F_i^j|$, and the errors in the reflectivity (dominated by laser fluctuations) $\Delta|R_i^j|/|R_i^j| \sim 0.03$, for $i = \mathrm{L}, \mathrm{R}$ and $j = +, -$. For the experiments on the nano-helices we find $\Delta|F_i^j| = 11\,\mu\mathrm{eV}$, and for the experiments on the AuNPs $\Delta|F_i^j| = 2\,\mu\mathrm{eV}$ (as one standard deviation).

These errors are then propagated on the corresponding quantities. For example, to calculate the error bars for g_α and g_σ, we used the following relationships. The error is calculated as $\Delta g = (dg/dA)\Delta A$, where $A = |\alpha_L/\alpha_R|$ for g_α, or $A = \sigma_L/\sigma_R$ for g_σ. Based on the definition of g, we find that $dg/dA = 4/(1+A)^2$ in both cases, so that $\Delta g = [4A/(1+A)^2](\Delta A/A)$. We then determine the relative error $\Delta A/A$ by independent error propagation from the quantities in A. For $A = \sigma_L/\sigma_R = |(F_\mathrm{R}^+ R_\mathrm{L}^+)/(F_\mathrm{L}^+ R_\mathrm{R}^+)|$ we have:

$$\Delta A/A = \sqrt{(\Delta|F_\mathrm{R}^+|/|F_\mathrm{R}^+|)^2 + (\Delta|R_\mathrm{L}^+|/|R_\mathrm{L}^+|)^2 + (|\Delta F_\mathrm{L}^+|/|F_\mathrm{L}^+|)^2 + (\Delta|R_\mathrm{R}^+|/|R_\mathrm{R}^+|)^2} \quad (1)$$

Similarly, for $A = \alpha_L/\alpha_R = |(F_\mathrm{L}^-/F_\mathrm{R}^-) \times (F_\mathrm{R}^+/F_\mathrm{L}^+)|$, we have:

$$\Delta A/A = \sqrt{(\Delta|F_\mathrm{L}^-|/|F_\mathrm{L}^-|)^2 + (\Delta|F_\mathrm{R}^-|/|F_\mathrm{R}^-|)^2 + (\Delta|F_\mathrm{R}^+|/|F_\mathrm{R}^+|)^2 + (\Delta|F_\mathrm{L}^+|/|F_\mathrm{L}^+|)^2} \quad (2)$$

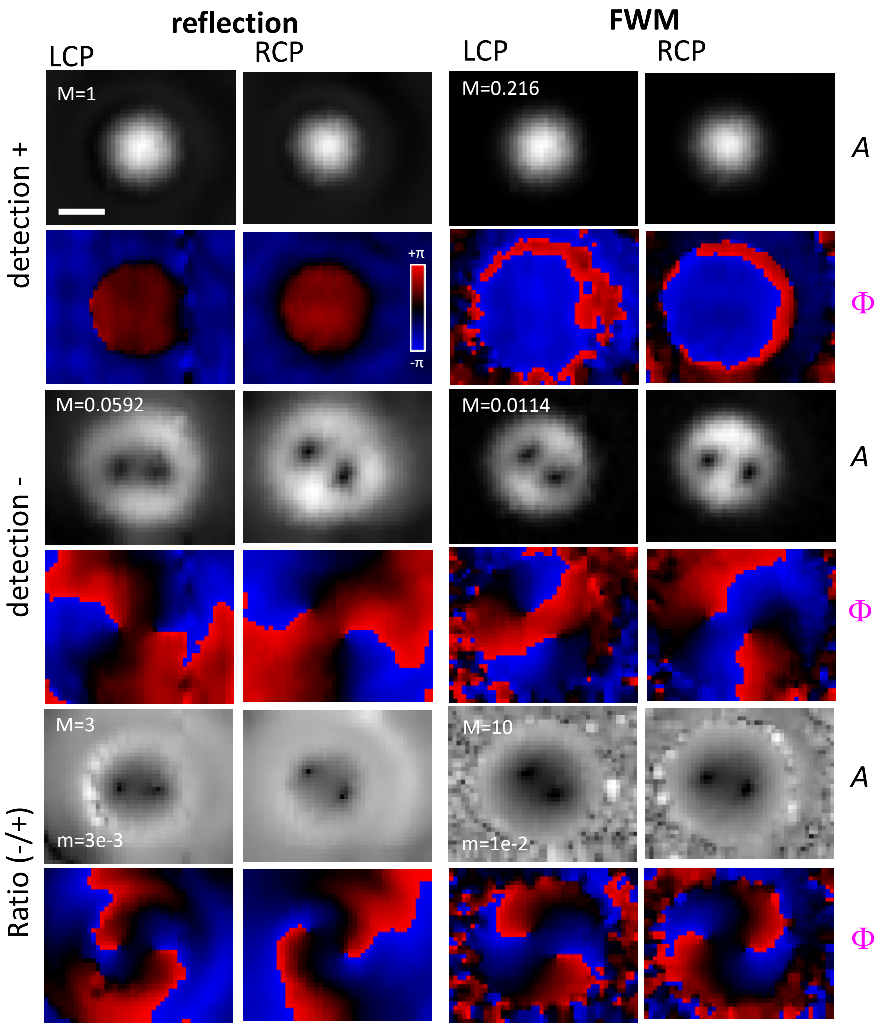

FIG. S1. In-plane (xy) images of the reflected probe and FWM field patterns (amplitude A and phase Φ) on an individual AuNP nominally spherical with 60 nm diameter, detected as co (+) and cross-circularly polarised component (-) relative to the input circularly polarised probe, comparing the cases of an RCP and LCP helicity of the incident circularly polarised probe, as indicated. The linear grey scale is from 0 to M for field amplitudes (A). Here, M=1 corresponds to a detected signal of 25 mV. The bottom row shows the ratio of the cross- relative to the co-polarised detected components, on a logarithmic grey scale from m to M as indicated. Phases (Φ) are plotted on a blue-black-red linear scale from $-\pi$ (blue) to π (red), as indicated. Measurements were performed using a 1.45NA oil immersion objective, with a pump (probe) power at the sample of 40 μW (20 μW). The in-plane step size was 19 nm, and the integration time per pixel was 1 ms. The probe followed the pump pulse with 0.5 ps delay. Scale bar: 200 nm.

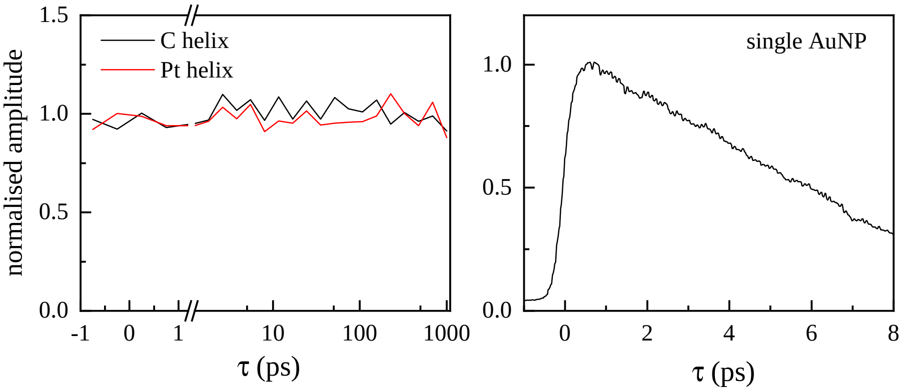

FIG. S2. Normalised FWM field amplitude co-polarised detected, measured at the nanoparticle focus center as a function of the pump-probe delay time. Left: single nanohelices, as indicated (specifically, these are C3 and Pt1). For these measurements the probe input polarisation was RCP, the pump (probe) power at the sample was 210 μW (22 μW) and the integration time per point was 1 ms. Right: single AuNP on nominally 60 nm diameter. Measurements were performed with an LCP input probe polarisation, using 40μW (20μW) pump (probe) power at the sample and 10 ms integration time per point.

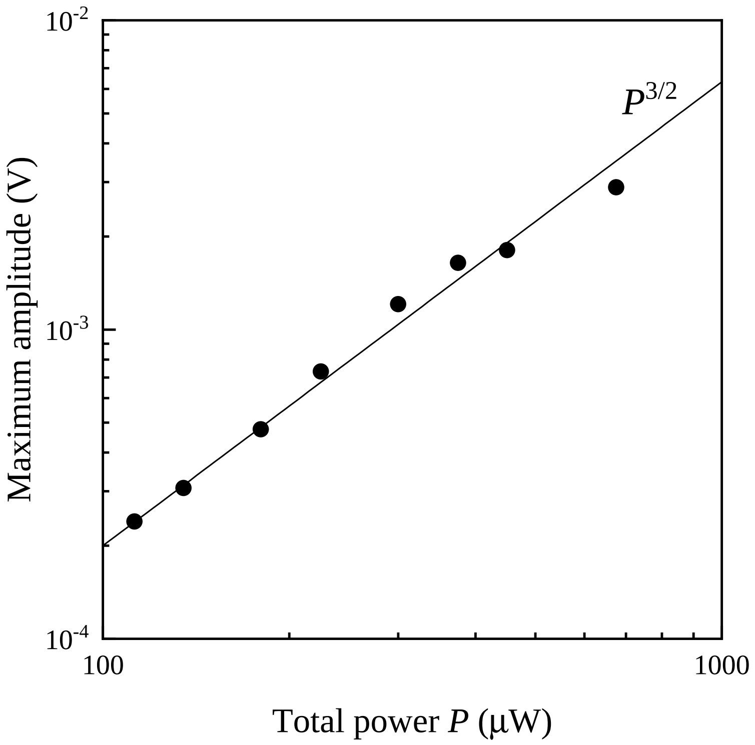

FIG. S3. FWM field amplitude co-polarised detected using an input LCP probe, measured on a Pt nano-helix test structure surrounded by water, with a 1.27 NA water immersion objective, as a function of the total (pump + probe) incident power (with a pump to probe power ratio kept to 2). The line shows the power dependence $P^{3/2}$ expected in the third-order regime.

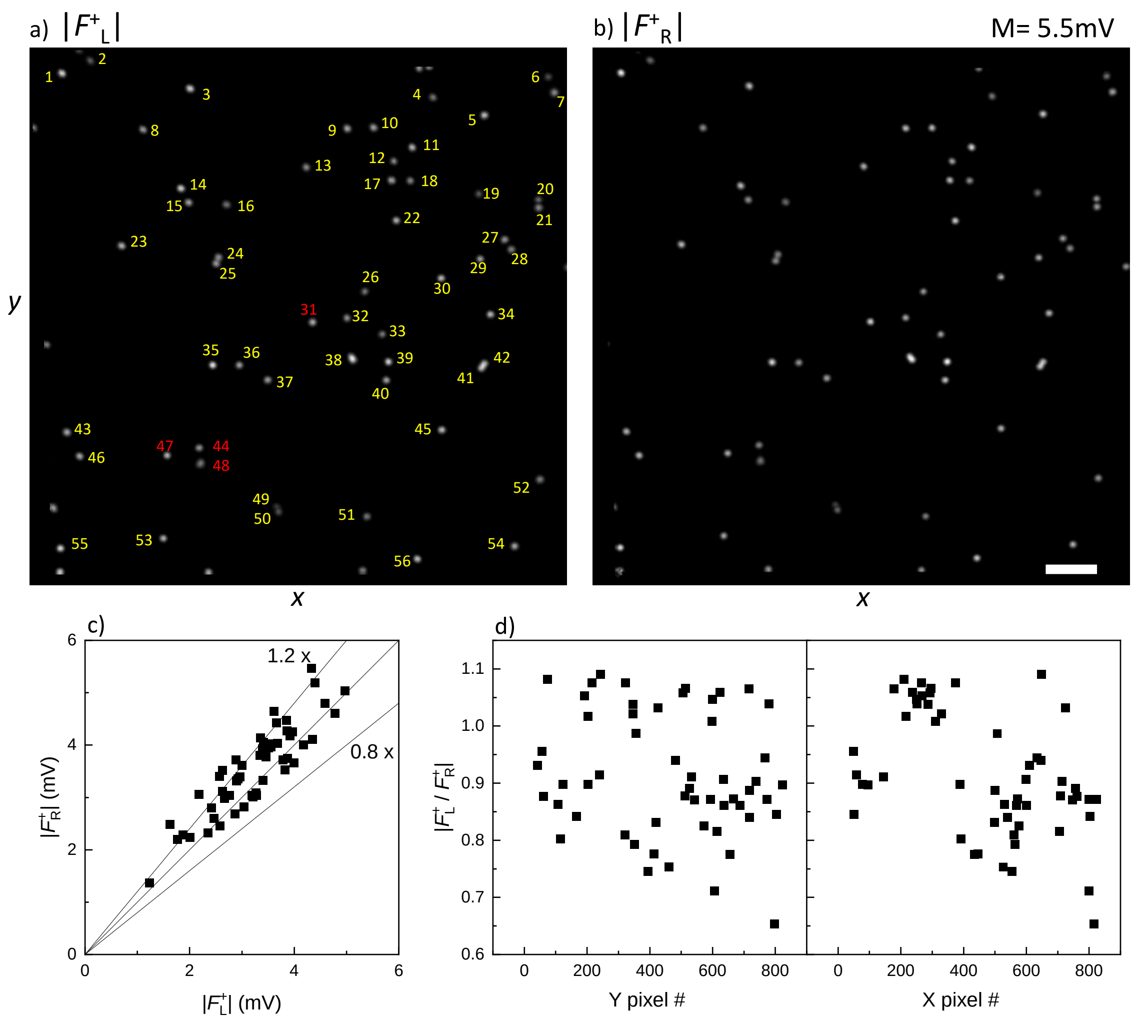

FIG. S4. Large overview in plane (xy) of the FWM field amplitude co-circularly polarised detected, for several individual AuNPs of 60 nm diameter deposited on glass, sequentially acquired using an LCP (a) and a RCP (b) probe input polarisation, as indicated. Measurements were performed using a 1.45NA oil immersion objective, with a pump (probe) power at the sample of 40 μW (20 μW). A 21 μm$\times$21 μm area was scanned in 25 nm steps in each direction, and the integration time per pixel was 0.5 ms. Scale bar: 2 μm. The linear grey scale is from from 0 (black) to M=5.5 mV (white). Nanoparticles indicated by the red numbers (44,47,48) are the same shown in the main paper Fig. 4. NP number 31 is the one shown in Fig. S1 and used the characterise the z dependence shown in Fig. S5. c) Peak amplitudes of the FWM field at the NP center, correlated to each other for each NP. d) The ratio of the peak amplitudes for each NP is plotted versus the pixel number of the sample stage movement in the x and y directions, showing no obvious correlation. The absence of an overall signal amplitude gradient from single particles indicates that no significant focus drift occurred during these large scans.

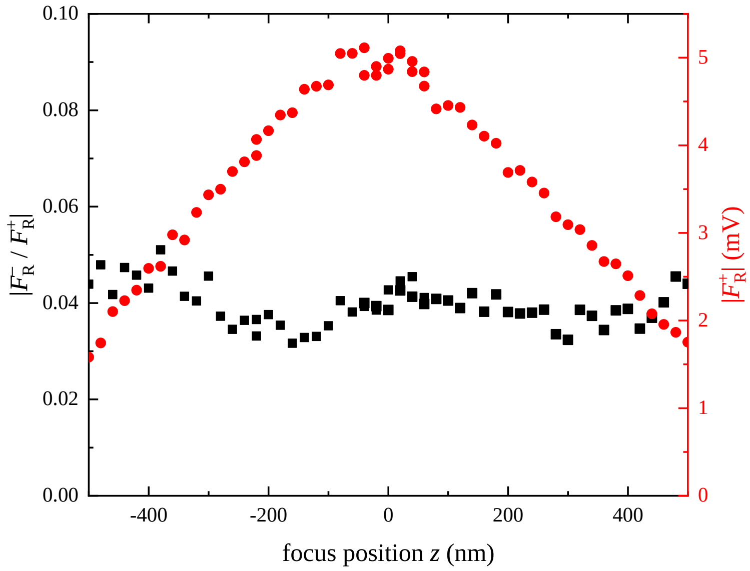

FIG. S5. Dependence of the FWM field amplitude on the axial focus position in the RCP incident probe configuration, on a AuNPs of 60 nm diameter deposited on glass, in the center of the large-area region of interest shown in Fig. S4, (NP number 31). The red points show the co-circularly polarised detected FWM field amplitude. The black points show the ratio between the cross- and co-polarised detected FWM field amplitudes, as indicated. The FWM ratio is found to be approximately constant as a function of the axial position over a $\sim$ 400 nm range around the optimum focus.

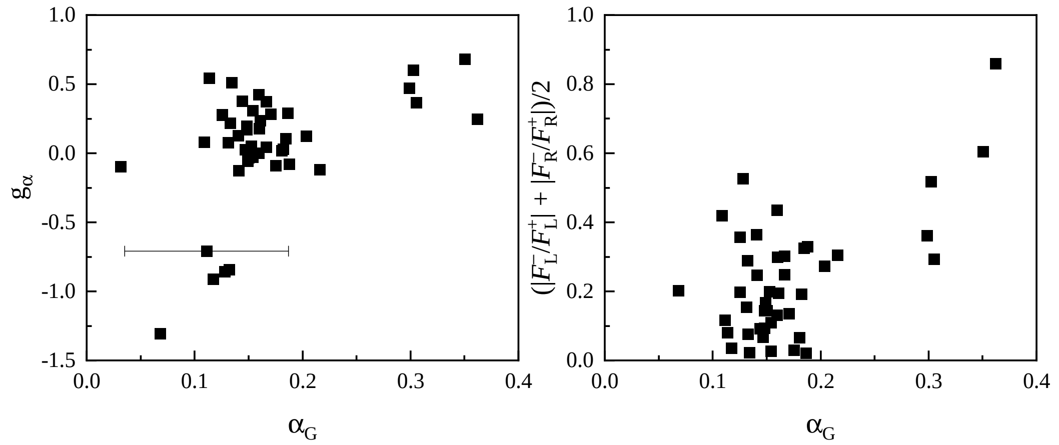

FIG. S6. Left: Chirality dissymmetry factor g_α on several individual AuNPs (same as in Fig. S4) versus in-plane ellipticity parameter α_G measured by wide-field optical extinction microscopy, using a band-pass filter centred at 530 nm wavelength of 43 nm width in the illumination beam path, and a rotatable linear polariser in the back focal plane of the condenser lens. The error bar in the ellipticity parameter shows the uncertainty on a representative point, obtained from repeating the fit of the measured cross-section versus polarizer angle due to the noise in the data [2,3]. Right: FWM amplitude ratio (average of L and R) versus in-plane ellipticity parameter. A positive correlation between the FWM amplitude ratio and in-plane ellipticity is observed for α_G significantly above uncertainty, $\alpha_G > 0.25$, consistent with our previous work [1].

REFERENCES

[1] Zoriniants, G., Masia, F., Giannakopoulou, N., Langbein, W. & Borri, P. Background-free 3D nanometric localization and sub-nm asymmetry detection of single plasmonic nanoparticles by four-wave mixing interferometry with optical vortices. *Phys. Rev. X* **7**, 041022 (2017).

[2] Payne, L. M., Langbein, W. & Borri, P. Polarization-resolved extinction and scattering cross-section of individual gold nanoparticles measured by wide-field microscopy on a large ensemble. *Appl. Phys. Lett.* **102**, 131107 (2013).

[3] Payne, L. M., Albrecht, W., Langbein, W. & Borri, P. The optical nanosizer - quantitative size and shape analysis of individual nanoparticles by high-throughput widefield extinction microscopy. *Nanoscale* **12**, 16215 –16228 (2020).
